# Supplementary material for: Macroscopic quantum tunnelling in spin filter ferromagnetic Josephson junctions
Source: Nat Commun. 2015 Jun 9;6:7376. doi: 10.1038/ncomms8376 (PMC4468849; doi:10.1038/ncomms8376)
Supplement: Supplementary Information — Supplementary Figures 1-3, Supplementary Note 1, Supplementary Discussion and Supplementary References [file ncomms8376-s1.pdf]

Supplementary Figures

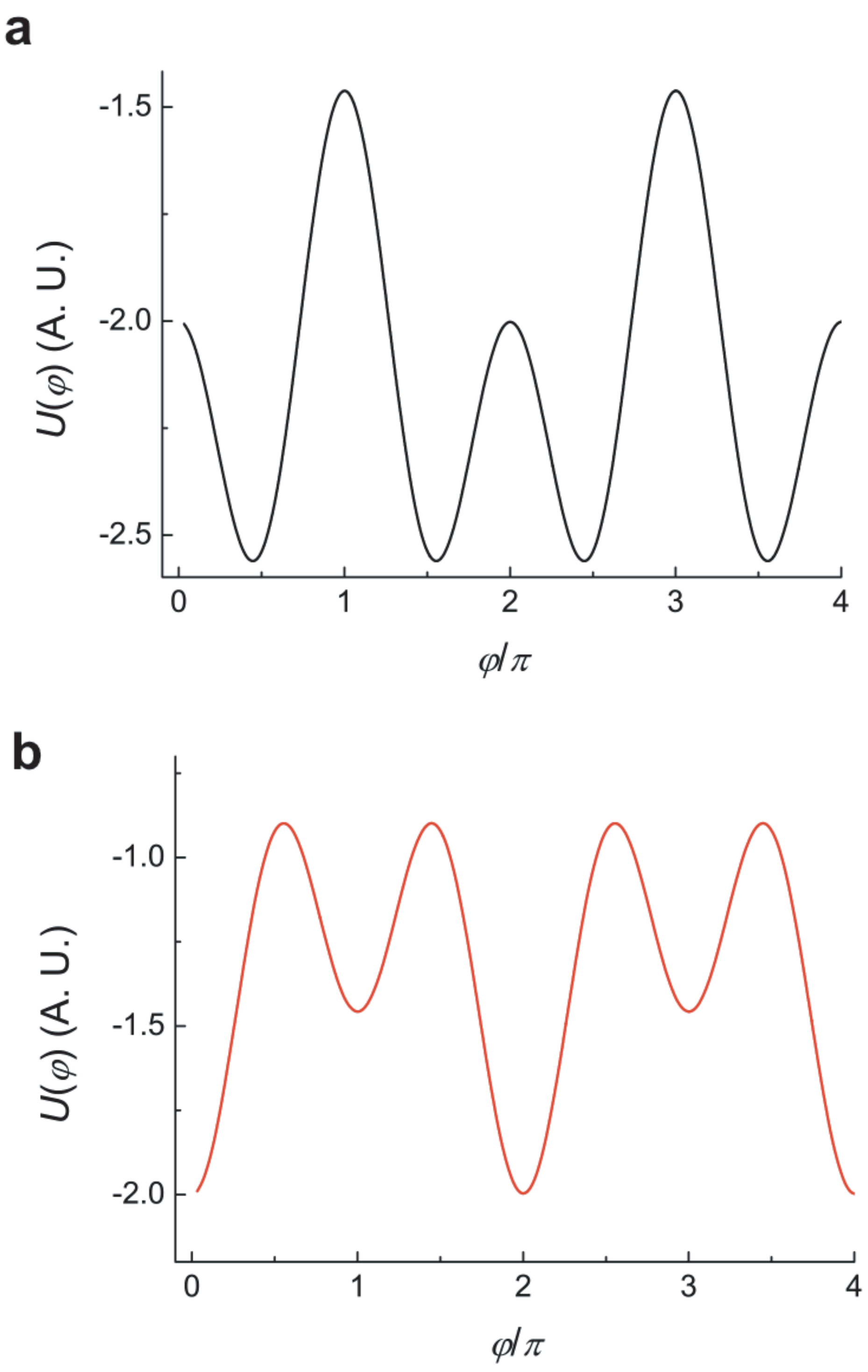

Supplementary Figure 1. Double well in the washboard potential. Washboard potential at zero bias current with a negative second harmonic component *g* (in panel **a**) and with a positive *g* (in panel **b**), respectively. In the figure $|g| = 2$.

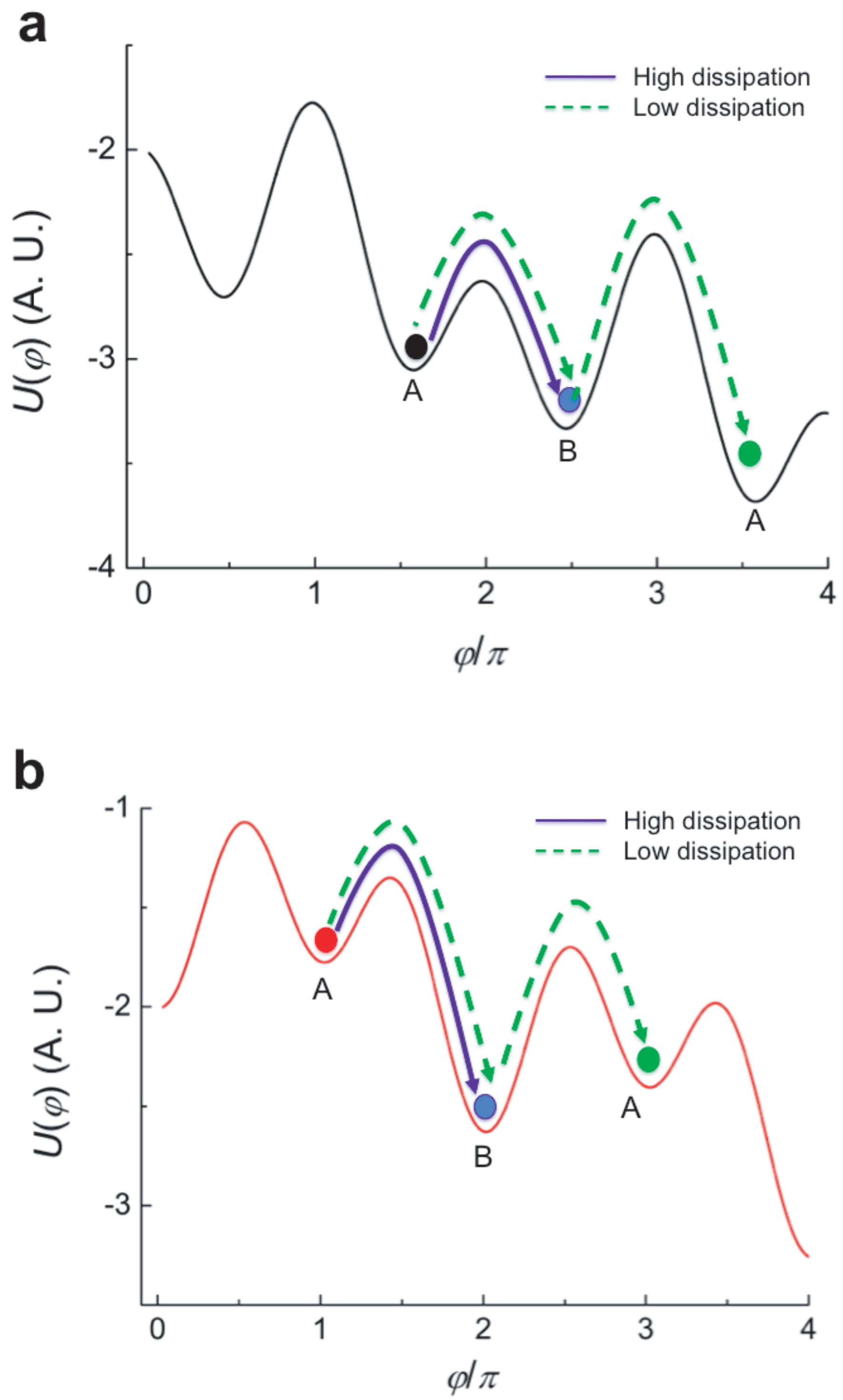

Supplementary Figure 2. Tilted double well washboard potential. Washboard potential for $g = -2$ (panel **a**) and for $g = 2$ (panel **b**), respectively, for values of the bias current close to the retrapping current. In both cases A and B mark the narrow and the wide potential well, respectively, when tilting the washboard from left to right. In case of high dissipation level (blue full line) the phase particle is retrapped in the well B (blue circle), while for low dissipation values (green dashed line) the phase particle can be retrapped in both wells with finite probability (green circle).

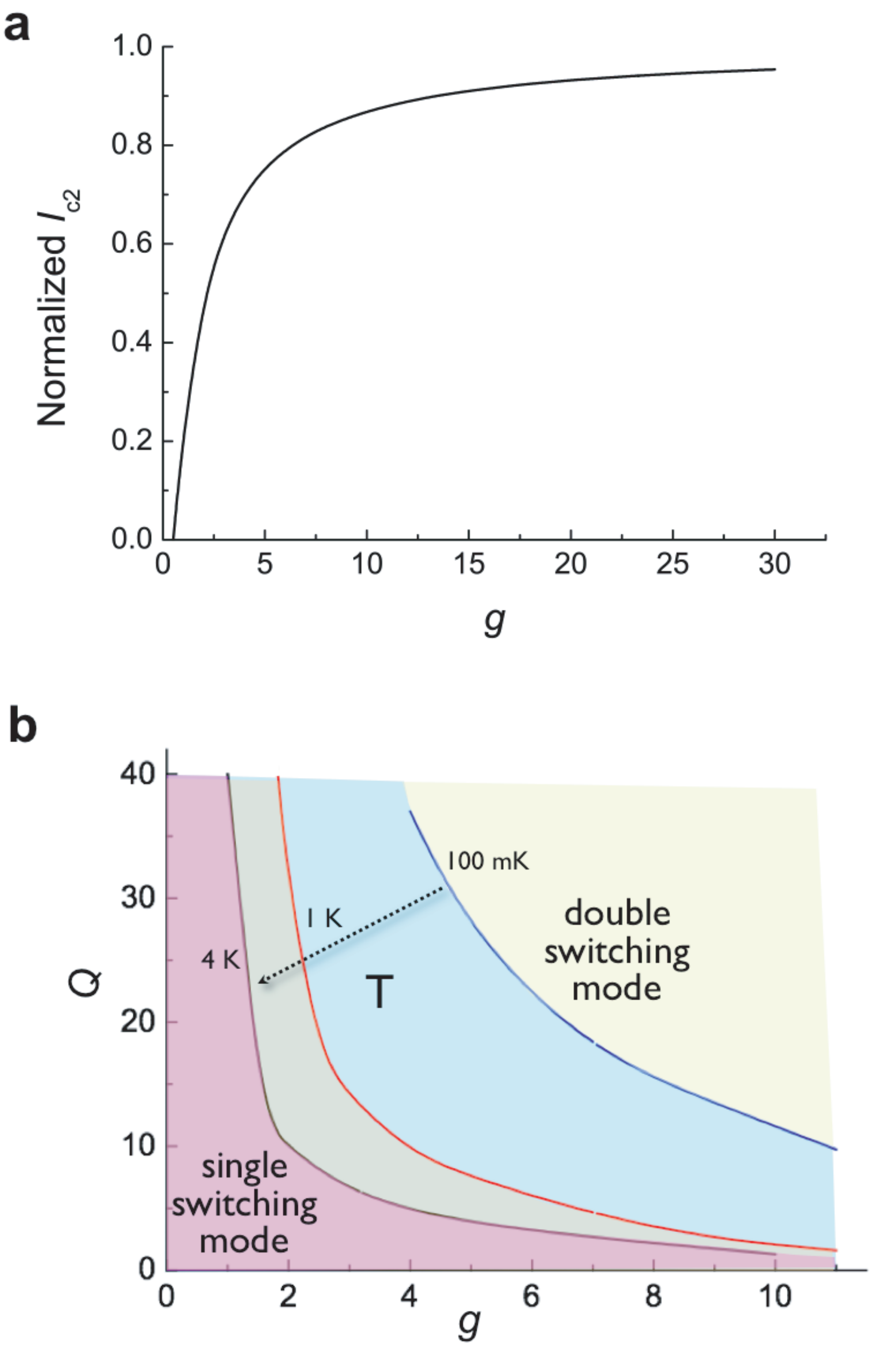

Supplementary Figure 3. Phase dynamics as a function of the g factor. (**a**) Critical current I_{c2} as a function of g, normalized to the critical current I_{c0}. (**b**) Phase separation between single and double mode switching distributions, for $g > 0$. These curves have been obtained from numerical simulations of the phase dynamics, by varying the g factor and the junction quality factor Q. Phase separation is temperature dependent, in the figure the curves corresponding to 4 K (black line), 1 K (red line) and 100 mK (blue line) have been reported. The dashed black arrow indicates the direction of temperature increasing. Below the phase separation curve at fixed temperature T, the switching distributions are unimodal, while they become bimodal above the phase separation curve.

Supplementary Notes

Supplementary Note 1. Second harmonic component in the current-phase relation: study of phase dynamics

In this section the study of phase dynamics will be addressed as a function of the temperature *T*, of the junction quality factor *Q* and more importantly of the relative amplitude between the first and the second harmonic components in the current-phase relation. The task will be undertaken through numerical simulations of the phase particle moving in the tilted washboard potential. The results achieved in this section are universal and apply to any type of Josephson system with a second harmonic component in the current phase relation.

In the framework of the Resistively and Capacitively Shunted Junction (RCSJ) model [1], the superconducting branch of the junction current-voltage characteristic corresponds to the confinement of the phase particle in one well of the washboard potential $U(\varphi) = -E_J\left(\cos\varphi + \varphi I/I_{c0}\right)$, where φ is the phase difference between the superconducting electrodes, $E_J = I_{c0}\hbar/2e$ is the Josephson energy and I_{c0} is the critical current in absence of thermal fluctuations. The escape from this metastable state corresponds to the appearance of a finite voltage across the junction and the phase particle runs down the tilted washboard potential with a damping $Q^{-1} = \left(\omega_p RC\right)^{-1}$, being ω_p the plasma frequency. When ramping the bias current *I*, the tilt of the potential increases and the height of the energy barrier between consecutive wells decreases.

The second harmonic component in the current-phase relation is measured by the $g = I_{c2}/I_{c1}$ parameter, explicitly appearing in the washboard potential $U(\varphi) = -E_1(\cos\varphi + g\cos 2\varphi)$, $E_1 = \hbar I_1/2e$. Effects are relevant in the phase dynamics for $|g| > 0.5$, while for $|g| < 0.5$ no secondary minima or maxima appears and the shape of the potential is only slightly modified [2]. The phase of the second harmonic component controls the location of the double well: if *g* is negative the double well sits in the middle of the potential maxima, where the minimum was located in absence of second harmonic component. The potential has two equal value minima and a secondary relative maximum (see Supplementary Fig. 1a). If *g* is positive the double well splits the maximum in a minimum creating a metastable minimum at a higher potential value, while all maxima retain the same value in this case (see Supplementary Fig. 1b). By increasing the bias current the double well structure in $U(\varphi)$ diagram tends to be washed out independently of the sign of *g*. When this occurs the secondary maximum or the metastable minimum disappears giving rise to a secondary escape event if the phase is initially localized in the narrower well marked by A in Supplementary Fig. 2 (we assume that the washboard is tilted from left to right). The current signaling the switching event ranges from 0 to I_{c0} depending on *g*.

From numerical simulations of phase dynamics, where we have prepared the phase particle in the position marked by A in Supplementary Fig. 2, the phase

particle unavoidably escapes from the smaller potential well. This occurs for any value of Q and in absence of thermal noise (T = 0 K). For small values of g and large dissipation an escape event cannot give rise to a running state, i.e., the particle simply rolls down in the wider well marked by B. This occurs when g is slightly larger than 0.5, thus the secondary critical current I_{c2} is lower than the junction retrapping current.

For larger values of g, I_{c2} becomes larger and close to the value of the main critical current, as shown in Supplementary Fig. 3a. The escape event prepared in the state A is in this case followed by a running state, provided that the junction quality factor Q is above a threshold value [3]. Preparing the system in A or B (see Supplementary Fig. 2) will determine two distinct sets of switching events. For very large g values, i.e. $g \approx 100$, the washboard potential tends to have identical maxima and minima, therefore the escape events collected from the two critical currents tend to mix each other.

To fully decode the signatures of the second harmonic in switching hystograms, we have to consider the effect of dissipation Q and of the temperature T via thermal noise. In actual escape experiments preparation as shown above is not possible because typically the phase particle is retrapped in the potential by sweeping down the bias current. In the resistive state the phase particle rolls down the washboard potential, and by reducing progressively the tilt of the washboard the phase particle will be trapped in one potential well, A or B in Supplementary Fig. 2. The study of the retrapping process is crucial in order to distinguish in which conditions and for which junction parameters the switching distribution is expected to be bimodal [4]. A closer analysis shows that the process of retrapping is not the same for the two different signs of g.

In absence of thermal noise for $g < 0$, when the bias current becomes smaller than the retrapping current, the running state no longer exists. The phase particle is rolling down into the double well. Depending on dissipation, the phase particle can stop in both wells. If dissipation is large ($1 \leq Q \leq 3$) the phase particle falls in the wider well marked by B in Supplementary Fig. 2a because dissipation faster depletes its kinetic energy (see blue full line in Supplementary Fig. 2a). In case of small dissipation the energy can be sufficient to overcome the relative maximum and the phase particle is able to fall in the well marked by A in Supplementary Fig. 2a (see the green dashed line). Therefore for high values of Q the phase particle can be found in both wells marked by A and B in Supplementary Fig. 2a. In addition, the thermal noise also at very low T can influence the low dissipation plot giving (or subtracting) small amount of energy to the phase particle, in such way that it could be found in both wells at the end of the retrapping process [2–4]. Therefore the distribution of escape events for the negative sign of g is typically bimodal.

The same process cannot occur for $g > 0$ in absence of thermal noise, because the phase particle can only fall in the wider well marked by B in Supplementary Fig. 2b. Switching distributions for $g > 0$ are typically unimodal at T = 0 K. However, for $T > 0$ K the thermal noise could induce the phase

particle to overcome the maximum, so the phase particle is stochastically trapped in the metastable minimum (see Supplementary Fig. 2b, well A). For smaller values of g, $1 \leq g \leq 3$, this can only occur for very small dissipation levels and at relatively high temperature (in this range of g the secondary barrier is roughly from 20% to 60% of the main one). Indeed, for very small dissipation and at high temperatures the thermal noise stochastically gives the phase particle a hit to overcome the barrier separating the wells B and A in Supplementary Fig. 2b (see the green dashed line). In this case the distribution is bimodal. For large values of g, $g \geq 6$, the secondary barrier is 80% of main one, thus also if dissipation is large both wells can trap the phase particle due to casual hits from noise. Thus a bimodal distribution is expected to be dominant for large g because both the wells tend to be occupied with finite probability.

Supplementary Discussion

A schematic phase diagram illustrating the various regimes of single or double switching modes is reported in panel b of Supplementary Fig. 3 as a function of the main physical parameters for $g > 0$ at three different temperatures. Retrapping in the metastable well tends to be strongly suppressed at low temperatures (100 mK). Just above the plotted isotherm the distribution is bimodal, but the number of events from metastable minimum are quite small. For instance to reach 1% of escape events at $g = 7$, Q has to be increase up to 7 at 1 K and up to 25 at 100 mK.

The switching histograms measured on spin filter Josephson junctions are single mode distributions in the whole temperature range, as shown in Fig. 3. The numerical outcomes reported in Supplementary Fig. 3b suggest that a single mode distribution is consistent only with $g \leq 2$ or obviously with a pure second harmonic current phase relation. With a pure second harmonic current phase relation, the washboard potential is again a single well potential, and the resulting switching events are collected in a unimodal switching distribution. Since $g \leq 2$ is not consistent with the period of the magnetic pattern [5], a pure second harmonic is the only possible explanation.

Supplementary References

1] Barone, A. & Paternò, G. *Physics and Applications of the Josephson Effect* (John Wiley and Sons, 1982).
[2] Goldobin, E., Koelle, D., Kleiner, R. & Buzdin, A. I. Josephson junctions with second harmonic in the current-phase relation: Properties of φ junctions. *Phys. Rev. B* **76,** 224523 (2007).
[3] Sickinger, H. et al. Experimental evidence of a φ Josephson junction. *Phys. Rev. Lett.* **109,** 107002 (2012).
[4] Goldobin, E., Kleiner, R., Koelle, D. & Mints, R. G. Phase retrapping in a pointlike φ Josephson junction: the butterfly effect. *Phys. Rev. Lett.* **111,** 057004 (2013).
[5] Pal, A., Barber, Z. H., Robinson, J. W. A. & Blamire, M. G. Pure second harmonic current-phase relation in spin-filter Josephson junctions. *Nat. Commun.* **5,** 3340 (2014).
